# Analysis, Design, and Implementation of a User-Friendly Differential Privacy Application

**DOI:** 10.3390/s25051358

**Published:** 2025-02-23

**Authors:** Reynardo Tjhin, Muhammad Sajjad Akbar, Clement Canonne, Rabia Bashir

**Affiliations:** 1 School of Computer Science, Faculty of Engineering, J12—Computer Science Building, University of Sydney, Sydney, NSW 2050, Australia; rtjh9350@uni.sydney.edu.au (R.T.); clement.canonne@sydney.edu.au (C.C.); 2 Centre for Health Informatics, Australian Institute of Health Innovation, Faculty of Medicine, Health, and Human Sciences, Macquarie University, Sydney, NSW 2113, Australia; rabia.bashir@mq.edu.au

**Keywords:** anonymization, privacy, differential privacy, machine learning, AI, security, application

## Abstract

In the era of artificial intelligence, ensuring privacy in publicly released data is critical to prevent linkage attacks that can reveal sensitive information about individuals. Differential privacy (DP) has emerged as a robust approach for safeguarding privacy, but its mathematical complexity often limits its accessibility to non-experts. This paper introduces a novel, user-friendly web application that bridges the gap between theoretical DP concepts and their practical application. The application includes two main features: a query version, which demonstrates DP mechanisms for statistical queries; and a privatize version, which applies DP techniques to entire datasets. A key contribution of this work is the identification of discrepancies in the implementation of maximum and minimum queries within the OpenDP library, revealing gaps between theory and practice. Additionally, this paper introduces a foundational framework for dataset privatization using OpenDP’s built-in methods. By providing an interactive platform, this work advances the public understanding of DP mechanisms and highlights areas for improvement in existing libraries. The application serves as both an educational tool and a step toward addressing practical challenges in the implementation of DP.

## 1. Introduction

Our era is marked by the rapid ascent of artificial intelligence (AI) and advanced computing, where technology permeates nearly every facet of our lives. Improving our daily routines increasingly involves integrating sophisticated technological solutions. Technology has transformed communication, enabling global connectivity, and it has streamlined tasks, enhancing speed and efficiency. This technology is currently advancing through the use of AI. The driving force behind AI’s impressive capabilities is not just complex algorithms, advanced engineering, or efficient programming, but also the vast amounts of data provided by humans. This data drives AI’s learning processes, enabling it to comprehend and tackle various tasks. To enrich user experiences, both companies and governments seek permission to gather data from users’ devices. In recent years, a sprawling landscape of partially interconnected databases has emerged, encompassing not only major players like Facebook and Google, but also thousands of other companies across industries, and these entities collect, analyze, acquire, share, trade, and utilize data on billions of individuals [1]. This data collection allows them to gain deeper insights into human behavior and more effectively target advertisements, often with the aim of maximizing profits.

However, the collection of individuals’ data can raise serious concerns, particularly in the event of a data breach. Misuse of such data by malicious actors can result in consequences ranging from social and financial harm to life-threatening situations. For instance, in 2018, Cambridge Analytica, a UK political consulting firm, unlawfully acquired personal data from nearly 87 million Facebook users, which were originally collected by a third party for academic research [2]. These data were then used to target U.S. voters with personalized political advertisements [3], affecting the social landscape of the U.S. election. Another alarming case with potentially life-threatening and financial repercussions occurred in August 2023, when the Police Service of Northern Ireland (PSNI) accidentally exposed sensitive personal information of 10,000 officers and civilian staff online. At the time, the organization was under threat from Northern Ireland-related terrorism, putting its members at serious risk of targeted attacks [4]. Consequently, the PSNI faced potential fines of up to GBP 750,000 [5]. Similarly, in April 2023, highly classified military documents were leaked online, including sensitive information regarding the war in Ukraine, Israel’s Mossad, and China’s interests in Nicaragua [6,7]. Such leaks can disrupt international strategies, pose national security risks, and alter global military operations.

Data play a critical role in enabling organizations to conduct analyses and research, fostering the development of improved products and services. As a result, robust security measures are imperative to ensure that sensitive data are not compromised. However, even with advanced security protocols in place, there is no absolute guarantee that a company’s systems will remain invulnerable to cyberattacks. Once breached, malicious actors may exploit the data, either by publishing them online or for financial or other motives. Without effective privacy-preserving mechanisms, such breaches can lead to the exposure of sensitive information. Furthermore, once data are disseminated online, it becomes exceedingly difficult to remove entirely, often leaving residual traces even after deletion efforts. Privacy-preserving mechanisms, therefore, serve as a crucial secondary safeguard when security measures are compromised. These mechanisms, such as data anonymization, ensure that, even in the event of a breach, individuals cannot be readily identified, thereby protecting their personal information. Additionally, legislative frameworks like the General Data Protection Regulation (GDPR) [8] in Europe have been enacted to enforce stringent standards for the ethical handling of personal data, emphasizing integrity and transparency.

In response to escalating privacy concerns, researchers have intensified their efforts to develop various privacy mechanisms to protect sensitive data. Data typically consist of headers, providing a brief description, and the actual information. While headers are generally innocuous, the information itself often contains sensitive data. Data lacking privacy protection can be linked with other public data, enabling the construction of detailed profiles that compromise privacy. An early method to safeguard data privacy was anonymization, involving the removal of personally identifiable information (PII) [9]. However, it was soon discovered that linking multiple tables or datasets could potentially re-identify individuals. Consequently, more advanced anonymization techniques, such as k-anonymity and its derivatives, have been developed to offer enhanced data privacy.

Companies are increasingly adopting differential privacy as a primary defense against privacy attacks. Many companies publish their research to demonstrate the applications of differential privacy [10,11,12]. For instance, Apple employs an epsilon parameter of two when collecting data on the most used health types in the HealthKit app [13]. Other companies like Google, LinkedIn, and Facebook are also implementing differential privacy to bolster their defenses against privacy breaches [13]. However, a knowledge gap exists between these companies and the general public. While differential privacy is recognized as an effective defense, the public lacks a clear understanding of its workings. This gap raises a crucial research question: how can we educate the general population on the current most effective method of defending against privacy attacks? To address this, we have developed a proof-of-concept application aimed at explaining how differential privacy functions in a straightforward manner.

Despite the widespread adoption of differential privacy (DP) by leading organizations, significant challenges remain in the domain of DP functions. These challenges include the inherent complexity of DP mechanisms, discrepancies between theoretical guarantees and practical implementations in existing libraries, and limited tools for dataset-level privatization. Additionally, the public and many practitioners struggle to understand DP’s inner workings, creating a knowledge gap that hinders broader adoption and effective application. This paper addresses these challenges by introducing a novel, user-friendly web-based application that bridges the gap between DP theory and practice. The key contributions of this work are as follows: 1. Accessible Education: The application provides an intuitive platform for users to understand DP mechanisms. It allows users to compare true and noisy outputs for statistical queries, offering a practical demonstration of privacy–utility trade-offs. 2. Novel Dataset Privatization Algorithm: A new algorithm is introduced to apply DP techniques to entire datasets, enabling users to privatize sensitive data before sharing. This algorithm ensures privacy for numerical attributes while addressing common pitfalls, such as nonsensical noisy outputs, through bounding and tweaking mechanisms. 3. Addressing Library Discrepancies: This work identifies and addresses inconsistencies in the implementation of DP functions in the OpenDP library, particularly for maximum and minimum queries, ensuring better alignment with theoretical models. 4. Real-World Applicability: The application supports practical experimentation by integrating real-world datasets, making it a valuable tool for both educational and applied purposes. This aligns with the broader goal of advancing public understanding and adoption of DP.

## 2. Literature Review

Privacy has emerged as one of the most important concerns in current society, particularly in the wake of the pervasive influence of the internet, where individuals can effortlessly upload vast amounts of personal data. However, without a comprehensive understanding of the underlying technologies, these data are susceptible to unwarranted collection and potential exposure to the public, paving the way for privacy breaches and attacks. To safeguard against such threats, various privacy defense mechanisms have been devised, including but not limited to *k*-anonymity, *l*-diversity, *t*-closeness, and, notably, the widely acclaimed concept of differential privacy. In the subsequent sections, we delve into the rationale behind the prominence of differential privacy and its pivotal role in fortifying our digital confidentiality.

### 2.1. k-Anonymity

*k*-anonymity is one of the earliest developed privacy defense mechanisms that protects individual data. A data release is known to provide *k*-anonymity if the information for each person contained in the release cannot be distinguished from at least *k* − 1 individuals whose information also appears in the release [14].

Ref. Sweeney [14] identified several attacks that can undermine the effectiveness of *k*-anonymity. The first is the unsorted matching attack, which assumes that the released information is unsorted. For instance, if there is already public information available and two subsequent datasets satisfying *k*-anonymity are released, adversaries can use the order of the data to infer individual identities, thus breaching privacy. This can be mitigated by randomly reordering the data before release. Another attack is the complementary release attack, where two publicly released datasets that each satisfy *k*-anonymity can be combined to form a larger table that may not meet *k*-anonymity, allowing for privacy breaches. To prevent this, subsequent releases must account for previously released information to ensure the combined dataset still satisfies *k*-anonymity. Finally, the temporal attack occurs when publicly released data are updated over time by adding, changing, or removing data. This allows adversaries to link new data with previously released information to reveal sensitive details. To mitigate this, the attributes of previously released data should be treated as quasi-identifiers, or subsequent releases should be based on the cumulative data to maintain *k*-anonymity.

### 2.2. l-Diversity

However, *k*-anonymity does not provide comprehensive protection against privacy attacks. Ref. Machanavajjhala et al. [15] introduced *l*-diversity to enhance the previous method, *k*-anonymity, in preventing privacy breaches. Ref. Machanavajjhala et al. [15] began by identifying privacy attacks that *k*-anonymity fails to defend against.

Compared to *k*-anonymity, *l*-diversity offers enhanced privacy without the need for the entire combined table. Additionally, *l*-diversity does not require the data publisher to possess as much information as the adversary. This is crucial when adversaries use background knowledge to infer sensitive data. With *l*-diversity, adversaries with background knowledge can only make inferences about the sensitive data they obtain. Since each adversary may draw different conclusions, *l*-diversity provides a stronger form of privacy by making it difficult for adversaries to distinguish one individual’s sensitive data from another’s.

### 2.3. t-Closeness

In order to further improve on *l*-diversity, ref. Li et al. [16] presented a new idea to reduce the limitations of *l*-diversity. Ref. Li et al. [16] first explored the limitations of *l*-diversity by presenting two different attacks that allow sensitive data being leaked to the public. The first attack is the skewness attack. Assuming we have a table with a single column of sensitive data, where the sensitive attribute has only two possible values, positive and negative, then suppose the probability of one value is significantly higher than the other. Next, consider a single $q^{*}$-block from the table, containing an equal distribution of positive and negative records. If this table is released, it satisfies the criteria for distinct 2-diversity but still poses a significant privacy risk. This is because each individual in the block would have a 50% probability of being either positive or negative compared to the actual population distribution. Therefore, despite the equal distribution within the block, the known overall higher probability of one value makes this block susceptible to privacy breaches. Another vulnerability is the similarity attack, where *l*-diversity fails to consider the semantic closeness of sensitive values. Even if these values can be generalized into a single sensitive attribute, it can still lead to significant privacy leakage. For instance, consider sensitive values related to health issues. If a target individual is in a $q^{*}$-block where all the sensitive values indicate stomach-related problems, the semantic closeness of these values allows an adversary to infer that the individual has a stomach-related issue, thus compromising their privacy.

When an adversary learns sensitive information about a person, there is a privacy breach on the sensitive data. This means that the adversary is able to gain more information about the person. The amount of information gained can therefore be used to measure the privacy the sensitive information has. Before the adversary learns the sensitive information, the adversary already has a prior belief about the sensitive attribute value of the individual. Upon learning the actual sensitive information, the adversary now has a posterior belief. Therefore, the difference between the posterior belief and the prior belief represents the amount of privacy the sensitive information has. Ref. Li et al. [16] separates this information gain into two parts: one about the whole population in the released dataset and another about specific individuals.

### 2.4. Differential Privacy

As we have seen in previous methods, each method is an extension of the other. For example, *l*-diversity is an extension of *k*-anonymity, and *t*-closeness is an improved extension of *l*-diversity. Each method is introduced in order to improve the current methods and to provide a stronger form of privacy with a stronger and more complete definition of privacy. On the other hand, differential privacy is not an extension of *t*-closeness. Ref. Dwork [17] mentioned that there are two natural models for privacy mechanisms: interactive and non-interactive. In the non-interactive setting, the data collector, or the entity responsible for ensuring privacy before releasing its information, will perform “sanitisation”, such as “anonymisation”, and “de-identification” on the dataset that contains private information. *k*-anonymity, *l*-diversity, and *t*-closeness fall under the non-interactive setting. In the interactive setting, the data collector creates an interface for the public to perform queries about the data, and the result may contain noise to ensure privacy. Differential privacy, therefore, falls under the interactive setting.

A concrete interactive privacy mechanism achieves ε-differential privacy by adding an appropriately chosen random noise to the true answer of a function *f*. The formal definition of differential privacy is as follows: A randomized function *K* gives ε-differential privacy if, for all datasets $D_{1}$ and $D_{2}$ that differ on at most one element, as well as all S ⊆ Range $\left(K\right)$, the following applies:

(1) $$Pr\left[K\left(D_{1}\right)\in S\right]\leq \exp\left(\varepsilon \right)\times Pr\left[K\left(D_{2}\right)\in S\right].$$

A mechanism K satisfies ε-differential privacy if, for all possible outcomes, the ratio of the probability of observing any particular outcome (when the mechanism operates on dataset $D_{1}$ to the probability of observing the same outcome when it operates on dataset $D_{2}$) differs by, at most, a factor of $e^{\varepsilon }$. Based on the equation above, $D_{1}$ and $D_{2}$ are neighboring datasets that differ by a single row of data. An adversary should not be able to identify an individual from an output if the output is ε-differentially private. Even if the participant removed her data from the dataset, no outputs would become significantly more or less likely [17].

Ref. Desfontaines [13] compiled a comprehensive list of real-world use cases of differential privacy (DP), illustrating the practical application of both pure ϵ-DP and approximate $\left(\epsilon ,\delta \right)$-DP. Table 1 summarize the key findings from their analysis.

- For companies implementing pure ϵ-DP, the ϵ values range from 0.1 to 16, with ϵ=2 being the most frequently used. The average ϵ value across these companies is approximately 4.39.
- In contrast, for those employing approximate DP ($\left(\epsilon ,\delta \right)$-DP), the ϵ values span a wider range, from 0.3 to 49.21. This broader range reflects a trade-off between privacy and data utility, allowing organizations to retain greater data utility while maintaining differential privacy guarantees. Importantly, the δ values used are very small, typically close to 0, ensuring that the probability of significant privacy loss remains low. The average ϵ value in this case is 13.17.

Based on these observations, a reasonable starting point for ϵ could be 2, with subsequent adjustments made after evaluating the data’s usability and privacy protection.

Differential privacy satisfies three important properties. The first property is the sequential property, which states that, if function $f_{1}\left(x\right)$ satisfies $\varepsilon_{1}$-differential privacy and function $f_{2}\left(x\right)$ satisfies $\varepsilon_{2}$-differential privacy, then the mechanism $g\left(x\right)=(f_{1}\left(x\right),f_{2}\left(x\right))$—which releases both results—satisfies $\left(\varepsilon_{1}+\varepsilon_{2}\right)$-differential privacy [18]. The second property is the parallel composition, which states that, if f(x) satisfies ε-differential privacy and we split the dataset *X* into *k* disjoint sets such that $x_{1}\cup \cdots \cup x_{k}=X$, then the mechanism that releases all of the results $f\left(x_{1}\right),\ldots ,f\left(x_{k}\right)$ satisfies ε-differential privacy. Finally, the last property is the invariance to post-processing, which states that if f(x) satisfies ε-differential privacy, then, for any randomized function *g*, applying function *g* to the result of f(x) also satisfies ε-differential privacy.

### 2.5. Comparison Across Privacy Mechanisms

As discussed earlier, *k*-anonymity has several limitations. It is ineffective against homogeneity attacks, where all sensitive values within a group are identical, and background knowledge attacks, where adversaries use external information to deduce sensitive data [19]. Additionally, ref. Meyerson and Williams [20] demonstrated that achieving optimal *k*-anonymity is NP-hard, meaning that determining the best method to generalize or suppress data for *k*-anonymity becomes computationally infeasible for large datasets. Moreover, *k*-anonymity often results in excessive suppression or generalization of data, leading to significant information loss, which compromises the utility of the anonymized dataset for tasks like data mining or analysis [21].

Similarly, *l*-diversity also has vulnerabilities, particularly to skewness and similarity attacks, where sensitive attributes are not diverse enough within a group to prevent inference [19]. On the other hand, *t*-closeness, while offering improvements, also has its challenges. One of its drawbacks is the complexity of understanding the relationship between the *t*-value and the information gained by an attacker. This is because it relies on the Earth Mover’s Distance (EMD) metric to measure the distance between distributions [19]. The literature proved that, for any constant *t* such that 0≤t≤1, finding the optimal *t*-closeness generalization is NP-hard, similar to the challenge in *k*-anonymity. Furthermore, these definitions—*k*-anonymity, *l*-diversity, and *t*-closeness—are not capable of preventing background knowledge attacks. In such scenarios, an adversary could potentially know all but one record in the dataset, making it easy to deduce the remaining sensitive information [22].

As we move further into the era of artificial intelligence, the reliance on large datasets for training AI models becomes increasingly critical. However, traditional privacy-preserving techniques, such as *k*-anonymity, *l*-diversity, and *t*-closeness, can disrupt the relationships between attributes in the data. This disruption can cause AI models to learn inaccurate or misleading patterns, leading to poor generalization and incorrect inferences. In contrast, differential privacy offers a mathematical guarantee of privacy, ensuring that data can be safely used without compromising utility. Differential privacy’s flexibility allows it to be adapted for modern applications, particularly in AI. For instance, the Gaussian mechanism, a key differential privacy technique, has been successfully implemented in deep learning frameworks to protect individual data while maintaining model accuracy [23].

### 2.6. Further Information About Differential Privacy

The following paragraphs provide a detailed explanation of differential privacy. We will begin by exploring its fundamental mechanisms, including the Laplace mechanism and Gaussian mechanism. Subsequently, we will delve into popular variants such as zero-concentrated differential privacy and Renyi differential privacy, highlighting their advantages over specific mechanisms. Additionally, we will discuss local differential privacy, address current vulnerabilities in differential privacy, and showcase the current solutions. Given the focus of this paper on the privacy of statistical releases, we will not delve into differential privacy that is related to machine learning or similar areas.

#### 2.6.1. Sensitivity

Before we start on the mechanisms of differential privacy, we must first understand the sensitivity of a function.

The $l_{p}$-sensitivity refers to the maximum possible change in the function output (measured using the $l_{p}$ norm) when a single record is added or deleted from the input [24]. Our definition of $l_{p}$-sensitivity is as follows: Let *f* be a query mapping from the space of datasets to $R^{k}$. Let *N* be the set of all possible pairs of neighboring datasets, i.e., $N=\{\left(D,D^{\prime }\right)|$*D* and $D^{\prime }$ are neighbors}. For a fixed positive scalar p, the $l_{p}$-sensitivity of *f* is defined by

(2) $$S\left(f;p\right)=max_{D,D^{\prime }\in N}{∥f\left(D\right)-f\left(D^{\prime }\right)∥}_{p}.$$

The sensitivity defined here is global as it is determined by the worst-case pair of neighboring datasets rather than the specific algorithm. For example, a query *f* that counts the number of data points in a dataset will have a sensitivity of 1, as the maximum change in the output of the query is 1 when a data point is added or removed. However, for other queries, sensitivity may be infinite if there are no bounds on the data. One approach to address this issue is clipping, where the entries in the dataset (or the output of the query) are limited to fall within a specified range [25].

#### 2.6.2. Laplace Mechanism

The Laplace distribution is defined by the probability density function $g\left(x|b\right)=\frac{1}{2b}exp\left(\frac{\left|x\right|}{b}\right)$, where x∈R. The parameter *b* is the scale parameter and the variance of the distribution is $2b^{2}$. The Laplace mechanism operates by adding calibrated noise sampled from a Laplace distribution to the output of a query $f\left(D\right)\in R^{k}$, producing a noisy version of the result. The variance of the noise is adjusted to match the $l_{1}$-sensitivity of the function f(D). The Laplace mechanism can be formally defined as follows [24]:

(3) $$A_{L}\left(D;f,\varepsilon \right):=f\left(D\right)+\left(Z_{1},Z_{2},\cdots ,Z_{k}\right),\;Z_{i}\overset{\mathrm{ind}.}{∼}\mathrm{Laplace}\left(S\left(f;1\right)/\varepsilon \right)\forall i.$$

The Laplace mechanism is guaranteed to be ε-DP [18]. A higher value of ε corresponds to a stronger privacy guarantee as it entails adding larger noise to the query output.

#### 2.6.3. Gaussian Mechanism

However, we do not need to use a strong privacy guarantee on publicly released information all of the time. A stronger privacy guarantee means a lower utility and, sometimes, this is unwanted. Therefore, a relaxed version of pure ε-differential privacy was introduced—the approximate differential privacy [26]. Approximate-DP introduces a new term δ. This term controls the strength of the relaxation of differential privacy. The smaller the value of δ, the stronger the privacy guarantee. The formal definition of (ε,δ)-DP is as follows: Let ε and δ≤1 be two non-negative scalars. A mechanism A is (ε,δ)-differentially private if, for any two neighboring datasets $D_{1}$ and $D_{2}$, as well as for any S⊆Range(A), the following applies:

(4) $$\mathrm{Pr}\left[A\left(D_{1}\right)\in S\right]\leq \exp\left(\varepsilon \right)\;\cdot \;\mathrm{Pr}\left[A\left(D_{2}\right)\in S\right]+\delta .$$

One of the mechanisms that satisfy (ε,δ) being differentially private is the Gaussian mechanism. The Gaussian mechanism samples noise from a normal distribution with a mean of 0. While the Gaussian mechanism does not guarantee ε-differential privacy, it can ensure $\left(\varepsilon ,\delta \right)$-differential privacy. This mechanism is commonly used in machine learning, such as in DP-Stochastic Gradient Descent (or DP-SGD). The Gaussian distribution is defined by the probability density function $g\left(x\right)=\frac{1}{\sigma \sqrt{2\pi }}e^{-\frac{1}{2}{\left(\frac{x-\mu }{\sigma }\right)}^{2}}$. The parameter σ is determined by $\sigma =S\left(f;2\right)\sqrt{2\ln1.25/\delta }$ [18], where S(f;2) is the $l_{2}$-sensitivity. It is important to note that while the Laplace mechanism uses $l_{1}$-sensitivity, the Gaussian mechanism uses $l_{2}$-sensitivity.

The Gaussian mechanism employs noise with less variance than the Laplace mechanism. This is due to the fact that ${∥x∥}_{2}\leq {∥x∥}_{1}$, which implies that the $l_{2}$-sensitivity will always be ≤$l_{1}$-sensitivity [25]. As a result, the noise sampled from the normal distribution has a lower variance.

#### 2.6.4. Variants of Differential Privacy

As previously mentioned, the common use case of approximate DP is in machine learning where multiple “queries” are performed with the same privacy budget. Composition bounds using approximate DP provides an even looser bound for advanced composition [24]. Therefore, approximate DP with a tighter bound for advanced composition was introduced, which includes the following: Renyi differential privacy (RDP) and Zero-concentrated differential privacy (zCDP).

**Renyi Differential Privacy**: Ref. Mironov [27] defined the concept of differential privacy based on the concept of Rényi divergence. The definition of Rényi divergence is as follows: For two probability distributions, *P* and *Q*, which are defined over *R*, the Rényi divergence of order α>1 is

(5) $$D_{\alpha }\left(P||Q\right)≜\frac{1}{\alpha -1}\log E_{x∼Q}{\left(\frac{P\left(x\right)}{Q\left(x\right)}\right)}^{\alpha }.$$

As α approaches 1, the Rényi divergence $D_{1}\left(P||Q\right)$ converges to the Kullback–Leibler (KL) divergence, which is also known as relative entropy:

(6) $$D_{1}\left(P||Q\right)=E_{x∼P}\log\frac{P\left(x\right)}{Q\left(x\right)}.$$

As α increases to +∞, Rényi Divergence becomes related to max divergence, focusing on the most extreme event, i.e., the largest ratio between $P\left(x\right)$ and $Q\left(x\right)$). Specifically,

(7) $$D_{\infty }\left(P||Q\right)=\underset{x\in \mathrm{supp}Q}{\sup}\log\frac{P\left(x\right)}{Q\left(x\right)}.$$

Furthermore, a randomized mechanism *f* is ϵ-differentially private if and only if its distribution over any two adjacent inputs *D* and $D^{\prime }$ satisfies

(8) $$D_{\infty }\left(f\left(D\right)\left|\right|f\left(D^{\prime }\right)\right)\leq \epsilon .$$

Therefore, Rényi divergence is when α=+∞ closely corresponds to differential privacy. Hence, the formal definition of RDP is as follows:

A randomised mechanism f:D→R is said to have ε-Renyi differential privacy of order α, or (α,ε)-RDP for short, if for any adjacent *D*, $D^{\prime }\in D$ it holds that

(9) $$D_{\infty }\left(f\left(D\right)||f\left(D^{\prime }\right)\right)\leq \varepsilon .$$

If *f* is an (α,ε)-RDP mechanism, it also satisfies (ε+(log1/δ)/(α−1),δ)-differential privacy for any 0<δ<1. The basic mechanism that achieves RDP is the Gaussian mechanism. For a function *f* with $l_{2}$ sensitivity, the following mechanism satisfies (α,ε)-RDP [25]:

(10) $$F\left(x\right)=f\left(x\right)+N\left(0,\sigma^{2}\right)\;\mathrm{where}\;\sigma^{2}=\frac{{\left(l_{2}-\mathrm{Sensitivity}\right)}^{2}\;\cdot \;\alpha }{\left(2\;\cdot \;\varepsilon \right)}.$$

**Zero-Concentrated Differential Privacy**: Ref. Bun and Steinke [28] used the work of Dwork and Rothblum [29] as a starting point to introduce the concept of zero concentrated differential privacy (zCDP). The definition of zCDP uses the Renyi divergence between probability distributions as a different method of capturing the requirement that the privacy loss random variable is sub-Gaussian [28]. The formal definition of zCDP is as follows: a randomized mechanism $M:X^{n}\to Y$ is (ξ,ρ)-zero-concentrated differentially private ((ξ,ρ)-zCDP henceforth) if, for all $x,x^{\prime }\in X^{n}$ differing on a single entry and all α∈(1,∞), the following applies:

(11) $$D_{\infty }\left(M\left(x\right)||M\left(x^{\prime }\right)\right)\leq \xi +\rho \alpha ,$$

where $D_{\alpha }\left(M\left(x\right)||M\left(x^{\prime }\right)\right)$ is the α-Renyi divergence between the distribution of M(x) and the distribution of M(x’).

We define ρ-zCDP to be (0,ρ)-zCDP. Equivalently, we can replace (11) with

(12) $$E\left[e^{(\alpha -1)Z}\right]\leq e^{\left(\alpha -1)(\xi +\rho \alpha \right)},$$

where Z=PrivLoss(M(x)||M(x’)) is the privacy loss random variable. The value *Z* represents how well we can distinguish *x* from x’, given the output M(x) or M(x’) only. If Z>0, the observed output *M* is more likely to be derived from the input *x* than x’, and vice versa. On the other hand, if Z=0, the observed output *M* is equally likely to be derived from both the inputs *x* and x’.

A mechanism $M:X^{n}\to Y$ is ε-differentially private if and only if P[Z>ε]=0, where Z=PrivLoss(M(x)||M(x’)) is the privacy loss of M on the arbitrary inputs *x*, $x’\in X^{n}$ differing in one entry. On the other hand, *M* being (ε,δ)-differentially private is equivalent, up to a small loss in parameters, to the requirement that P[Z>ε]≤δ.

In contrast, zCDP entails a bound on the moment generating function of the privacy loss *Z*, that is, $E\left[e^{(\alpha -1)Z}\right]$ as a function of α−1. Intuitively, this means that *Z* resembles a Gaussian distribution with mean ξ+ρ and variance 2ρ. In particular, we obtained strong tail bounds on *Z*. Namely, (12) implies that

(13) $$P\left[Z>\lambda +\xi +\rho \right]\leq e^{\left(-\lambda^{2}/4\rho \right)}$$

for all λ>0.7.

Therefore, zCDP mandates that the privacy loss random variable is centered near zero. In other words, *Z* remains “small” with high probability, making larger deviations from zero increasingly unlikely. As a result, distinguishing between the inputs *x* and x’ based on the output of M(x) or M(x’) becomes difficult. It is important to note that the randomness in the privacy loss is solely due to the randomness within the mechanism *M*.

zCDP imposes a stricter requirement than RDP by limiting the Rényi divergence across various orders. However, the constraint becomes less strict as α increases [25]. zCDP is also similar to RDP in that the Gaussian mechanism can be employed as the foundational mechanism. Specifically, for a function $f:D\to R^{k}$ with $l_{2}$ with $l_{2}$ sensitivity, the following mechanism ensures ρ-zCDP [25]:

(14) $$F\left(x\right)=f\left(x\right)+N\left(0,\sigma^{2}\right)\;\mathrm{where}\;\sigma^{2}=\frac{{\left(l_{2}\;-\;\mathrm{Sensitivity}\right)}^{2}}{2\;\cdot \;\rho }.$$

#### 2.6.5. Local Differential Privacy

When individuals do not trust a central aggregator (or the central unit that stores data) with their data, they can add calibrated noise to their data from their own devices. This concept, known as local differential privacy [30], is based on the notion of differential privacy. In this approach, before transmitting their data to the central node, individuals perturb their data with noise, ensuring that the mechanism used satisfies local differential privacy.

Our definition of local differential privacy is as follows: Let ε be a positive scalar. A mechanism A guarantees ε-local differential privacy if for any two values $x_{1}$ and $x_{2}$, and for any S ⊆ Range $\left(A\right)$, we have

(15) $$Pr\left[A\left(x_{1}\right)\in S\right]\leq exp\left(\varepsilon \right)\times Pr\left[A\left(x_{2}\right)\in S\right].$$

#### 2.6.6. Current Issues in DP and Its Solutions

One of the possible attacks on differential privacy is on the least significant bits when the noise is generated. Due to the representation of decimals as floating-point numbers in computers, they are not always accurately represented. Instantiating floating-point implementation of the Laplacian mechanism without appropriate defense mechanisms will not be able to satisfy ϵ-differential privacy if it misses some output values on input *D* and others on input $D^{’}$ [31].

In order to combat this attack, some authors have proposed using discrete distributions instead of continuous distributions. One approach includes developing an additive noise mechanism that operates on integers as much as possible, allowing one to then rely on simple properties of IEEE floating-point standards to argue durability against numerical attacks [32]. Another work uses mechanisms that satisfy approximate-DP using discrete Gaussian distribution [33]. Another approach to defend against the floating-point attack is using a snapping mechanism where the noisy output is carefully truncated and rounded. Nevertheless, this potentially reduces the utility of the output. However, ref. Jin et al. [34] mentioned that although these mechanisms defend against floating-point attacks, they are still susceptible to another attack, which is the timing side channel attack. An adversary that observes the time it takes to draw a sample can determine the generated noise’s magnitude. This reveals the noise contained in the noisy result and, thus, also reveals the private value [34]. Ref. Jin et al. [34] mentioned that, to defend against timing side channel attacks, the code must be run in a constant time execution.

## 3. Literature Findings

The main findings in the literature are as follows:

- There are two natural models for privacy-preserving mechanisms: non-interactive methods, such as anonymization techniques like *k*-anonymity, *l*-diversity, and *t*-closeness; and interactive methods, such as differential privacy [17].
- Differential privacy is currently widely used in the fields of machine learning and artificial intelligence, where ensuring privacy in data is crucial.
- While differential privacy offers strong privacy guarantees, it is not immune to certain vulnerabilities, such as floating-point precision attacks.
- Differential privacy is a vast field with different mechanisms and variants that satisfy differential privacy.
- Differential privacy has real-world applications, including the United States Census Bureau’s use in post-secondary employment outcomes, as well as implementations by companies like Facebook, Apple, LinkedIn, and Google [13].

While privacy is crucial, it is equally important for users to comprehend the fundamental mechanisms behind privacy protection. However, the concept of differential privacy is inherently complex, involving advanced mathematical principles that are not easily accessible to the general public. To bridge this gap, this paper aims to develop a user-friendly application that demonstrates the application of differential privacy in a statistical context. This tool will not only utilize the sophisticated privacy libraries, but also provide an intuitive interface to help users understand and appreciate the underlying principles of differential privacy. By doing so, we hope to make the concept more approachable and foster greater adoption and trust in privacy-preserving technologies.

### 3.1. An Additional Feature: A Privatizing Dataset

Moreover, differential privacy is not only applicable to data statistical releases, but it also plays a crucial role in artificial intelligence, where sensitive data are often used to train models. Despite its importance, to the best of our knowledge, there currently appears to be no comprehensive method for privatizing a dataset before its release. Privatizing a dataset is critical not only for training artificial intelligence models, but also to prevent the leakage of sensitive data. For example, the Netflix Prize dataset, which contains anonymized movie ratings from 500,000 Netflix subscribers, was re-identified by [35], who were able to link the Netflix records to known users and uncover potentially sensitive information, including apparent political preferences. Similarly, in March 2014, blogger Chris Whong used FOIL to obtain detailed 2013 NYC taxi trip data, including fares, trip details, and GPS coordinates, which were linked by hashed identifiers. The md5 hash function used for these identifiers was cracked, leading to the re-identification of the entire 50 GB dataset. These re-identified data were then used to determine how much celebrities had tipped by linking them to specific taxi trips [36]. These examples highlight the urgent need for robust methods to privatize datasets before their release to ensure the protection of sensitive information.

There are existing methods to privatize user data, such as local differential privacy. Local differential privacy allows for the privatization of user data on their devices before it is sent to a central server, making it suitable for scenarios where users do not trust the central server. However, even if the central server is not fully trusted, a robust approach is still needed to ensure privacy before releasing the dataset. Additionally, anonymization methods like k-anonymity are commonly used for this purpose, but they are vulnerable to de-anonymization attacks, which can compromise data privacy. To address this need, our application includes a method for privatizing an entire dataset, ensuring that sensitive information remains protected throughout its life cycle.

### 3.2. Tools to Use Differential Privacy

In developing an application based on differential privacy, we explored various differential privacy libraries, including Google’s DP libraries, PyTorch’s Opacus, SecretFlow, IBM’s Differential Privacy, TensorFlow Privacy, OpenDP, and OpenMined/PyDP [37]. Since our application focuses on statistical releases, we considered libraries that support differential privacy in this context. We narrowed our options to Google’s DP libraries, IBM’s Differential Privacy, OpenDP, and OpenMined/PyDP. We ultimately chose OpenDP (https://docs.opendp.org/en/stable/index.html (accessed on 24 June 2024)) due to its active community and the library’s overarching goals. OpenDP offers a flexible framework for implementing differential privacy, allowing users to select different mechanisms as needed. Additionally, OpenDP has a broader definition of privacy that encompasses essential components that are often overlooked by other libraries. The extensive documentation provided by OpenDP is also invaluable for user education. The most compelling reason for our choice is the active and inclusive community, which encourages public contributions and innovation. With these factors in mind, we selected OpenDP as the backend for differential privacy in our application.

### 3.3. Contributions

The contributions of this paper are as follows:

- A straightforward proof-of-concept web application designed to illustrate the practical application of differential privacy, helping users to understand key concepts through interactive queries and real-world datasets.
- A foundation for future work in dataset privatization, where the new method introduced demonstrates how entire datasets can be privatized using mechanisms that satisfy differential privacy.

## 4. Methods

In this section, we focus on the application, which has two distinct versions: query and privatize. The query version is intended for public use to demonstrate how differential privacy is currently applied. Differential privacy is primarily utilized in statistical database queries and in the fields of artificial intelligence, including machine learning and deep learning. Given the frequent publication of statistical data releases, differential privacy is commonly employed to protect privacy in these cases. On the other hand, the privatize version is an additional feature designed to privatize the entire dataset before publication.

### 4.1. Dataset

We employed two distinct datasets to evaluate the application: the Angiographic Coronary Disease dataset [38] and the Adult Census Income dataset [39]. The Adult Census Income dataset was primarily utilized to test the query version of the application, focusing on assessing the results after applying differential privacy to individual queries. Conversely, the Angiographic Coronary Disease dataset was used to evaluate the “privatize” version of the application, where the entire dataset was subjected to differential privacy mechanisms. The evaluation methods and corresponding results will be discussed in the next chapter. It is important to note that distinguishing between categorical and numerical attributes is crucial for the privatization algorithm (as detailed in Section 4.2.5). The algorithm effectively operates on numerical attributes, whereas categorical attributes remain unaffected by the process.

#### 4.1.1. Angiographic Coronary Disease

The Angiographic Coronary Disease dataset originates from the clinical and non-invasive test results of 303 patients who underwent angiography at the Cleveland Clinic in Cleveland, Ohio. The dataset was donated on 30 June 1988 [38]. While the original database contains 76 attributes, the version used in this study comprises only 14 attributes. Personal identifiers, such as names and social security numbers, were removed and replaced with dummy values to ensure anonymity. This dataset is primarily used to train machine learning algorithms to detect the presence of heart disease in patients, with the target attribute representing an integer value ranging from 0 (indicating no presence of heart disease) to 4 (indicating various stages of severity).

The dataset includes five numerical attributes: age, trestbps, chol, thalach, and oldpeak. These are included alongside eight categorical attributes: sex, cp, fbs, restecg, exang, slope, ca, and thal. Only the numerical attributes are subject to the privatization process. The target attribute, “num,” is categorical and contains four distinct categories.

#### 4.1.2. Adult Census Income

The Adult Census Income dataset was extracted from the 1994 U.S. Census Bureau database by Ronny Kohavi and Barry Becker (Data Mining and Visualization, Silicon Graphics) [39]. This dataset consists of 14 attributes, with the final column indicating whether an individual’s income exceeds 50,000. It is widely used in machine learning for predictive tasks. While there are five numerical attributes in the original dataset (fnlwgt, education.num, capital.gain, capital.loss, and hours.per.week), our method, explained in Section 4.2.2, automates the classification of attributes as categorical or numerical. Due to this method, only one numerical attribute, “fnlwgt”, is recognized by the application, while the others are treated as categorical. The predicted attribute, “income”, is binary and consists of two categories: ≤50 k and >50 k.

### 4.2. Application

The web application was built with Python as the backend, utilizing OpenDP as the main library for implementing differential privacy. Flask was chosen for handling web pages and web tasks due to its lightweight nature and its design, which facilitates quick and easy initial development while also allowing for scalability to support more complex applications. HTML, CSS, Jinja template, and JavaScript were used to develop the frontend part of the application to ensure a responsive and user-friendly interface.

**Figure 1:**
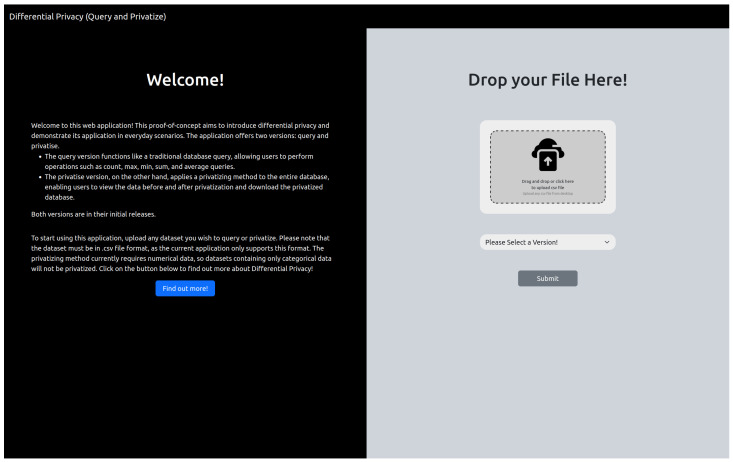
Homepage.

Upon accessing the webpage, users encounter a layout with two distinct sections: the left side presents instructions and definitions aimed at introducing differential privacy to the public, while the right side offers a platform for users to upload their preferred database (Figure 1). Currently, the application exclusively accepts files in “.csv” format, which are comma-separated values files. A “.csv” file features the first line as the header, labeling the column data, followed by the data itself in subsequent lines, where each row represents a single data entry. After uploading their dataset, users can select from the available versions. The default version is the “query” version.

Following selection, users can proceed to the next page by clicking the submit button.

#### 4.2.1. “Query” Version

If the user selects the “query” version, the user will arrive at the “query” page. This page follows the same design as the home page. The left side of the page provides an instruction on how to use the page, the meaning behind each parameter, how differential privacy works, and the meaning behind the outputs. On the right side, the user can select five different aggregate functions: count, max, min, sum, and average. For each aggregate function, the user can input different values for each parameter. The query will return both the true values and the differentially private results, allowing the user to compare the differences. Each query will be explained in greater detail in the next few paragraphs.

#### 4.2.2. “Count” Function

The “count” aggregate function incorporates an “epsilon” value, which dictates the level of privacy the output should maintain. A lower epsilon value indicates higher privacy, resulting in an output further from the true value due to increased noise generation. Typically, the epsilon value is set to 1.

The “count” aggregate function supports both numerical and categorical data. For numerical data, the function offers flexibility by accepting “maximum value” and “minimum value” values, allowing users to count rows falling within specified numerical ranges. Categorical data can be counted based on selected categories, with the application automatically identifying categorical columns without manual input from the user. To determine if a column is categorical, the application calculates the number of unique values and the total number of data points, and it then computes the absolute difference between these values. If the ratio of the difference to the total number of data points exceeds a certain threshold (currently set at 0.9), the data are classified as categorical [40]:

(16) $$\mathrm{data}\;\mathrm{is}\;\mathrm{categorical}\;\mathrm{if}\;\frac{N-n_{\mathrm{unique}\;\mathrm{values}}}{N}\geq \mathrm{threshold},$$

where *N* represents the total number of data points in the dataset and $n_{\mathrm{unique}\;\mathrm{values}}$ represents the number of unique values in the dataset. While this automated approach streamlines data classification, it may occasionally overlook data falling below the threshold. As a result, most of the data in the Adult Census Income dataset is identified as categorical due to the dataset’s large size.

The “count” query utilizes the Laplace mechanism, which generates random noise based on the Laplace distribution using a specified scale parameter. The scale is calculated as 1 divided by the epsilon value, determining the amount of noise added to the true count.

(17) $$\mathrm{scale}=\frac{1}{\mathrm{epsilon}}\;\mathrm{where}\;\mathrm{Sensitivity}\;\mathrm{of}\;\mathrm{Function}\;=\;1.$$

The sensitivity of this mechanism is 1, as the addition or removal of a single row from the dataset can change the count by, at most, 1. Therefore, the noise generated is added to the true count to produce a noisy output that is ε-differentially private. Since counting inherently involves integer values, the resulting output is also ensured to be an integer, mitigating the risk of floating-point attacks, as discussed in the literature review. The OpenDP library requires specifying the “contribution” variable, which represents the influence a single row of data can have on other rows. Presently, users are not required to provide a value for this variable as it is set to 1 by default.

The following paragraphs will discuss the other queries: max, min, sum, and average. These queries require numerical data; hence, they cannot be applied to categorical data. If categorical data are detected, the application will display an error message stating “This data is categorical and cannot be used for this query”.

#### 4.2.3. “Maximum”, “Minimum”, and “Sum” Functions

The “maximum”, “minimum”, and “sum” queries will be discussed together here as they share a similar approach for obtaining differentially private results. Similar to the “count” query, these operations also require users to input an “epsilon” value. However, in addition to an epsilon value, these queries also accept “lower bound” and “upper bound” values to define the range of the data within which the query is executed.

The “maximum” and “minimum” queries locate the rows containing the actual maximum and minimum data values, respectively. To obtain the differentially private result, the “sum” function is applied to that single data point and then combined with calibrated noise. In contrast, the “sum” function computes the actual result to which the calibrated noise is added to release a differentially private sum.

This query also employs the Laplace mechanism, which necessitates determining the sensitivity of the function. This sensitivity calculation requires the “lower bound” and “upper bound” values of the function to be provided as they dictate the potential impact of new data added to the dataset. Without proper technique, this process can quickly become unreliable. To address this, the application implements a clipping technique [25], requiring the input of “lower bound” and “upper bound” values. Additionally, the query utilizes symmetric distance to compute the sensitivity of the following function [41]:

(18) $$\begin{array}{cc} \mathrm{Sensitivity}\;\mathrm{using}\;\mathrm{Symmetric}\;\mathrm{Distance} & =d_{Sym}\left(u,v\right)\\ \; & =\sum_{x}\left|\#\left\{i:x=u_{i}\right\}-\#\left\{i:x=v_{i}\right\}\right|. \end{array}$$

The scale for the Laplace mechanism is then determined by dividing the sensitivity by the epsilon value provided by the user. The scale can be written using the following formula:

(19) $$\mathrm{scale}=\mathrm{contribution}\;\cdot \;\frac{l_{p}-\mathrm{sensitivity}}{\varepsilon },$$

where the contribution is set to 1 by default, and the $l_{p}-\mathrm{sensitivity}$ is calculated using the symmetric distance (Equation (18)).

Depending on the epsilon value, the resulting output may significantly deviate from the true value; larger epsilon values yield outputs closer to the truth. To mitigate numerical attacks, the resulting output matches the numerical data type.

#### 4.2.4. “Average” Function

Similar to the queries mentioned before, the “average” query requires an “epsilon” value from users. Additionally, akin to the “max”, “min”, and “sum” queries, the “average” query also accommodates “lower bound” and “upper bound” values. Another essential input is the “constant” value, which is unique to the OpenDP library. This “constant” is necessary for downsampling or upsampling, as per the requirements of the “count” query, which will be discussed further.

Instead of directly calculating the true average and adding calibrated noise, the differentially private “average” query leverages the sequential property of differential privacy. This query combines a “sum” query and a “count” query. As demonstrated earlier, the differentially private “sum” and “count” queries can be computed separately and then divided to obtain the differentially private average—a method known as the plug-in approach in the differential privacy literature. However, OpenDP suggests that this approach may not yield the best results. Although it does provide a differentially private value, the utility is often lower compared to OpenDP’s solution. This is because the “plug-in” approach increases variance and introduces bias and asymmetry to the resulting distribution, which is particularly noticeable in smaller samples [42]. Therefore, for an “average” query, the “count” query must be executed first to determine the dataset’s size. The dataset is then adjusted to match the “count” query result. In cases of oversampling, where there are insufficient data, the “constant” value is used to fill the gaps. Subsequently, the differentially private “average” query can be performed.

This query also utilizes the Laplace mechanism. The sensitivity of the function can be similarly calculated to previous queries, and the scale is determined using the same formula (Equation (19)). The key difference with the “average” query is that it employs two differentially private queries. As a result, the “epsilon” value must be divided equally between them. Consequently, the “count” query receives half of the “epsilon” value provided by the user, and the “sum” query receives the other half.

#### 4.2.5. “Privatise” Version

**Figure 2:**
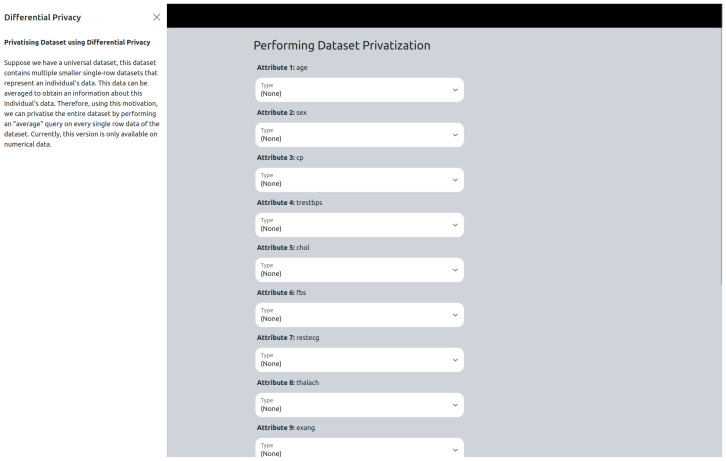
Privatize page.

**Figure 3:**
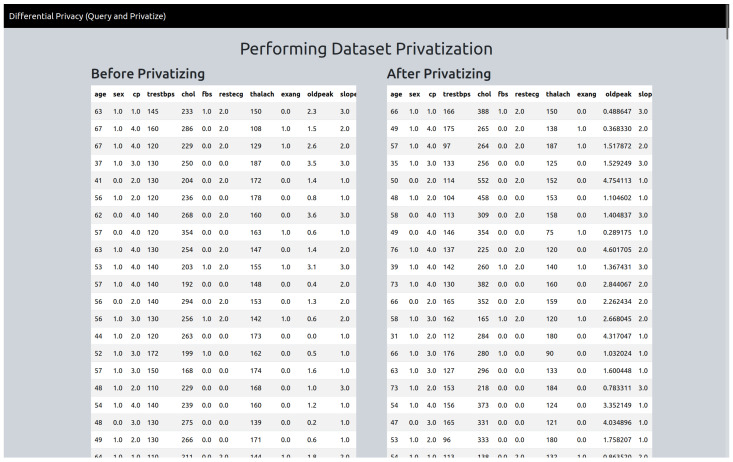
Privatize output page.

If the user selects the “privatise” version, they will be directed to the “privatise” page (Figure 2). Here, users can access instructions by clicking the instruction button, which opens an off-canvas page with detailed guidance. To proceed, users are required to input specific parameters for each numerical attribute, including epsilon values and constant values, as well as lower and upper bounds. Currently, categorical attributes are ignored, and differential privacy is only applied to numerical attributes. After privatizing the data, the user will be directed to the results page where the before privatizing (the original data) and after privatizing datasets are shown (Figure 3). The user is also able to download the privatized dataset, which will be in “csv” format.

The dataset privatization is handled by two primary algorithms. Algorithm 1 iterates through each row of the dataset and calls Algorithm 2, which performs the privatization using a differentially private average query. The main concept is that, for each row, Algorithm 2 applies a differentially private average query, using the Laplace mechanism, to each numerical attribute. In Algorithm 1, it first iterates over all columns in the dataset (Line 1), and it then checks if the attribute is numerical or categorical. For numerical columns, the algorithm iterates over each row (Lines 2 and 3), retrieves the numerical data, and calls Algorithm 2 to apply differential privacy based on the epsilon, constant, lower bound, and upper bound values input by the user. Currently, the Laplace mechanism is only implemented for numerical attributes, limiting the algorithm’s functionality on categorical data.

Statistical features such as maximum, minimum, mode, and average are commonly used to describe the characteristics of a dataset. In this approach, each data point is treated as a representative of itself. Through using a differentially private average query, we generate a privatized version of the average for each individual data point. This allows us to obscure the exact values, ensuring that an attacker cannot deduce the true value of any specific data point. This method effectively maintains the usefulness of the dataset while safeguarding it through differential privacy.
**Algorithm 1** Privatizing dataset**Input:** Dataset *D*, where *D* contains $c_{1},c_{2},\cdots ,c_{n}$, a list of ε values, lower bound, upper bound values, and constant values1:**for** $c_{i}$ in dataset *D* **do**2:    **if** $c_{i}$ is numerical **then**3:        **for** row $r_{j}$ in $c_{i}$ **do**4:           Obtain the data of column $c_{i}$ and row $r_{j}$5:           Perform an average query on the data based on ε value, lower bound, upper bound value, and the constant value6:           Update the dataset to the privatized value7:        **end for**8:    **end if**9:**end for**10: ** **11:**return** privatized dataset

In other words, a universal dataset *D* consists of multiple smaller subsets $D_{i}$. We assume that these smaller subsets $D_{i}$ are single-row datasets. Each single-row dataset $D_{i}$ may contain one or more columns $\left\{c_{1},c_{2},\cdots ,c_{n}\right\}$. By treating each single-row as a separate entire dataset, we can create a privatized average query to obtain the average value of the dataset.

The randomness in the differential privacy method to generate noise can create a non-sensible value. For example, with smaller values of epsilon, numerical data like age can return negative values or values that are too large to make sense. In order to ensure that the data are believable, the average query is tweaked such that it returns within the bounded values provided by the user (Algorithm 2). Based on Algorithm 2, Lines 2 to 4 ensure that the counting of the number of rows involved is greater than 0, and Lines 6 to 8 ensure that the mean is within the given bounds.
**Algorithm 2** Private Average Query**Require:** 
Sensitive Data *d***Require:** 
Bounds $\left(b_{0},b_{1}\right)$**Require:** 
Constant *c***Require:** 
Contribution contrib**Require:** 
Epsilon ε1:Instantiate **count** variable2:**while count** ≤ 0 **do**3:    obtain a differentially private count query4:**end while**5:Instantiate **mean** variable6:**while mean**$\leq b_{0}$ or **mean** $\geq b_{1}$ **do**7:    obtain a differentially private average query8:**end while**9:**return mean**

## 5. Experiments, Results, and Evaluation

This section explores the results obtained from using the application and implementing differential privacy with OpenDP. Additionally, we will outline the limitations of our work.

### 5.1. Epsilon Analysis

The “epsilon” parameter is crucial for achieving a private query result. Each query in this study was executed with the following varying epsilon values: 1, 2, 4, 10, 500, 1000, 2000, and 4000. The significance of these differences will be discussed later. The private count and private average queries were conducted using the Adult dataset, while the private max, private min, and private sum queries utilized the Angiographic Coronary Disease datasets. To provide a more extensive analysis of the epsilon values, each query was executed 100 times and the distribution of the outputs was evaluated.

**Figure 4:**
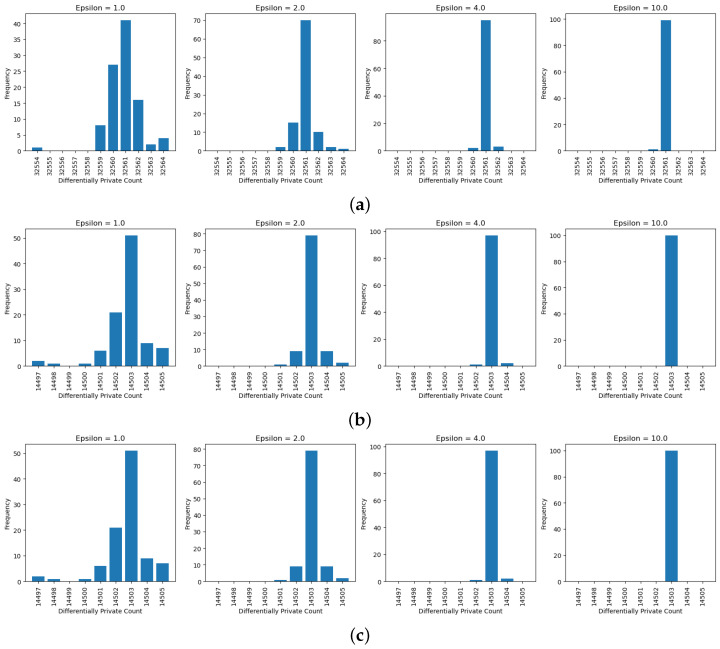
Private count epsilon analysis. (**a**) Private count analysis on the Adult Census Income Dataset, (**b**) private count analysis on the “fnlwgt” of the Adult Census Income Dataset, and (**c**) private count analysis on the “marital status” of the Adult Census Income Dataset.

**Figure 5:**
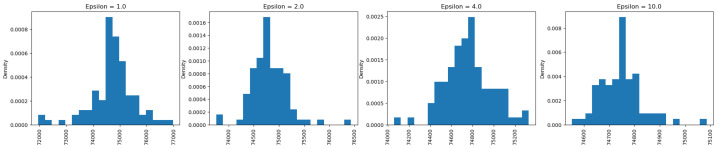
Private sum analysis on the “chol” of the Angiography Coronary Disease Dataset.

**Figure 6:**
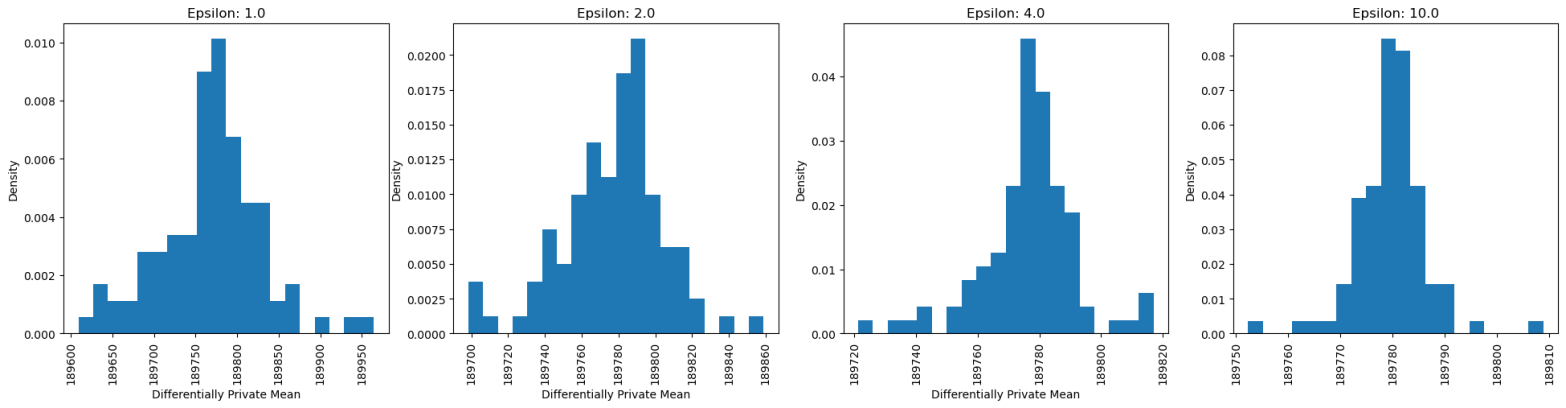
Private average analysis on the “fnlwgt” of the Adult Census Income Dataset.

**Figure 7:**
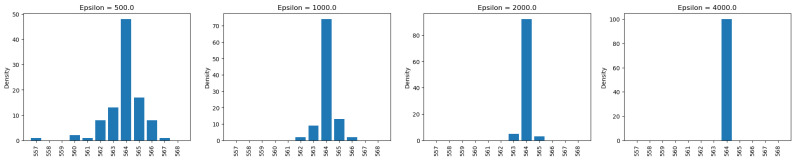
Private maximum analysis on the “chol” of the Angiography Coronary Disease Dataset.

**Figure 8:**
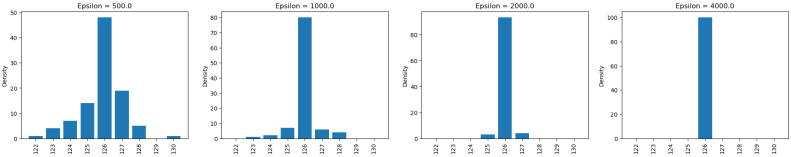
Private minimum analysis on the “chol” of the Angiography Coronary Disease Dataset.

After running 100 simulations with the epsilon set to 1, the output distribution resembles a bell shape, which is characteristic of the Laplace distribution used to generate the noise. This distribution has a pointy top, and adding this calibrated noise to the true value produces results similar to the Laplace distribution. As shown in the figures (Figure 4, Figure 5 and Figure 6), even with the epsilon value set to 1, the true value was the most frequently observed result for private counts, sums, and averages. As the epsilon value increased from 1 to 10, the true value appeared more frequently. For continuous results, based on Figure 5 and Figure 6, the truth value also appeared more frequently as it had the highest density. However, for the private maximum (Figure 7) and minimum (Figure 8) results, the output showed the same results but using very large epsilon values.

The private maximum and minimum queries utilize different epsilon values in order to obtain similar distributions to the other queries. Since OpenDP does not have built-in max or min functions, the private summation function is employed instead. The maximum and minimum values can be obtained by summing the single value, which is then added by a calibrated noise. Consequently, smaller epsilon values introduce excessive noise compared to other queries. As shown in Figure 7 and Figure 8, an epsilon value of 500 yields a distribution similar to an epsilon value of 1 in other queries. On the other hand, an epsilon value of 4000 returns mostly the true value. These large epsilon values suggest potential issues with the current implementation of private max and min functions.

When comparing against other use cases, it is preferable to use consistent epsilon values that produce similar output distributions to ensure a coherent understanding and interpretation of results based on epsilon values. Based on Desfontaines [13]’s blog post, we can conclude the following:

- Apple uses an epsilon value of ε=16 in their quick-type suggestions, which learns previously unknown words that are typed by sufficiently many users.
- Apple’s emoji suggestions finds the most popular emojis among users using an epsilon value of ε=4.
- Apple’s health type usage roughly calculates which health types are the most used in the HealthKit app using an epsilon value of ε=2.
- Apple’s Safari Energy Draining Domains and Safari Crashing Domains collect data on web domains to find which domains are the most likely to cause high energy consumption or crashes, respectively. Both of these features use a common epsilon value of ε=8.
- Facebook’s Movement Range Maps calculate the changes in the movement of Facebook users during the COVID-19 pandemic. It finds how much their users move during each day and how many people are generally staying at home. Each metric uses a daily value ε=1; hence, the overall privacy budget is ε=2.
- Israel’s Ministry of Health published a synthetic dataset of live births in 2014 in Israel using ε-DP with an epsilon value of ε=9.98, with singleton births (i.e., a single baby being born) as the privacy unit.

These findings indicate that it is uncommon to use ε values that are in the hundreds, suggesting possible inaccuracies in the implementations of the minimum and maximum query functions.

Looking at the continuous results for private sum and private average queries, the range of outputs changes as the epsilon value changes. Particularly, as the epsilon values increase, the difference between the largest output and the smallest output of the results decreases. Based on Figure 5, the range of output goes from 72,000 to a number slightly greater than 76,000 when the epsilon value is 1, while the range of output decreases as the range covers 74,500 to a number slightly greater than 75,900. This is also supported by the information shown in Figure 6, where the range of output covered from 189,600 to 190,000 when epsilon was 1 while the range of output covered from 189,750 to 189,810 when epsilon was 10. The main difference between the continuous and discrete outputs is that, in the continuous output, there are still some noises at epsilon that are equal to 10. However, for discrete outputs, the noises diminished significantly, therefore only leaving the truth value mainly. The main reason for this is that discrete values have a smaller range as they lack decimal points between them, making them easier to identify. In contrast, continuous values span a wide range, with even slight differences in decimal points resulting in distinct values.

Finally, it is important to consider the limitations and restrictions that differential privacy imposes. To our best knowledge, OpenDP uses int32 for discrete numbers, which means there is a maximum and minimum limit to integer outputs. The int32 type can represent values from −2,147,483,648 to 2,147,483,647. For numbers outside this range, a 64-bit integer (int64) should be used. However, when performing experiments with the private sum on the ’fnlwgt’ attribute, the output reached the maximum value of int32, suggesting that the implementation for int64 may not be supported. This could be due to the increased complexity associated with handling larger numbers. This observation is noteworthy and highlights a potential area for further development in OpenDP’s handling of large integers.

### 5.2. Contribution Analysis

In order to simplify things and to stick to the literature, we set the contribution value to 1 by default. In the literature, it is assumed that each row of data in the dataset contributes at most one row. However, OpenDP realizes that the influence of a single row of data can alter depending on the data. Therefore, OpenDP defines privacy as M(·), which is ε-differentially private at distance *k* if, for every pair of datasets *u* and *v* such that $d_{Sym}\left(u,v\right)\leq k$, we have that $D_{\mathrm{MaxDivergence}}\left(M(u),M(v)\right)\leq \varepsilon$ [41]. When comparing this to the literature, the definition of privacy refers to M(·) is ε-differentially private if, for every pair of adjacent datasets *u* and *v*, we have that $\mathrm{Pr}\left[M(u)\in S\right]\leq e^{\varepsilon }\;\cdot \;\mathrm{Pr}\left[M(v)\in S\right]$ [41]. Therefore, the definition or privacy differs by the distance between datasets. This section discusses and evaluates how the results differ when we include a contribution parameter.

**Figure 9:**
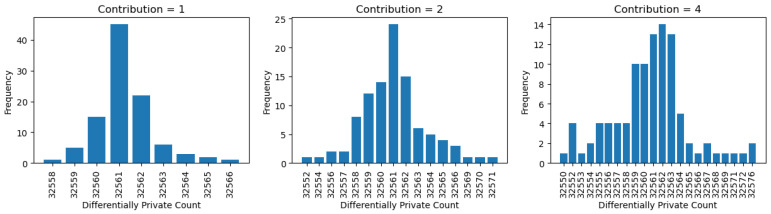
Contribution analysis on private count.

The concept of contribution defines how much influence each row in a dataset can exert, with a maximum impact on k rows. Our results (Figure 9) indicate that higher contributions necessitate greater privacy, resulting in more noise in the output. This relationship is inversely proportional to the epsilon value, where lower epsilon values correspond to higher privacy levels. The rationale behind this observation is that, as the contribution of an individual row increases, more effort is required to ensure that an adversary cannot distinguish between different individuals. Therefore, greater privacy protection is needed.

### 5.3. Bound Analysis

In order to calculate the sensitivity of a function, we need the lower and upper bounds of the data. In our experiments, we used three different sets of bounds for the cholesterol levels in the Angiographic Coronary Disease dataset. The first set of bounds is based on public knowledge: the lowest known cholesterol level is 93 mg/dL [43], and the highest is 982 mg/dL [44]. The second set is based on the dataset itself, with the lowest cholesterol level being 126 mg/dL and the highest cholesterol level in the dataset being 564 mg/dL. The third set considers potential future cholesterol levels, acknowledging that current known levels might change.

**Figure 10:**
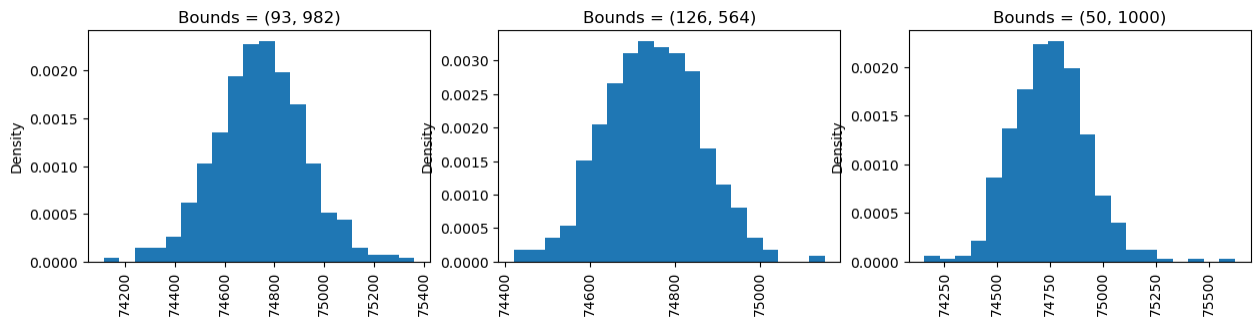
Bound analysis on private sum.

After running the simulations, the results in Figure 10 show minimal differences between the graphs. This is because we used symmetric distance to determine the sensitivity of the function (18), which depends on the largest absolute value within the bounds. Since the differences between the largest values of the three sets of bounds are not substantial (within 400 s), the generated noise does not vary significantly. However, a key consideration arises when using a smaller subset of the universal dataset. In such cases, public knowledge bounds should be used to prevent any privacy leakage. If an adversary knows the dataset’s bounds, they could infer sensitive information about the minimum and maximum values of the dataset.

### 5.4. Privatize Version Analysis

**Figure 11:**
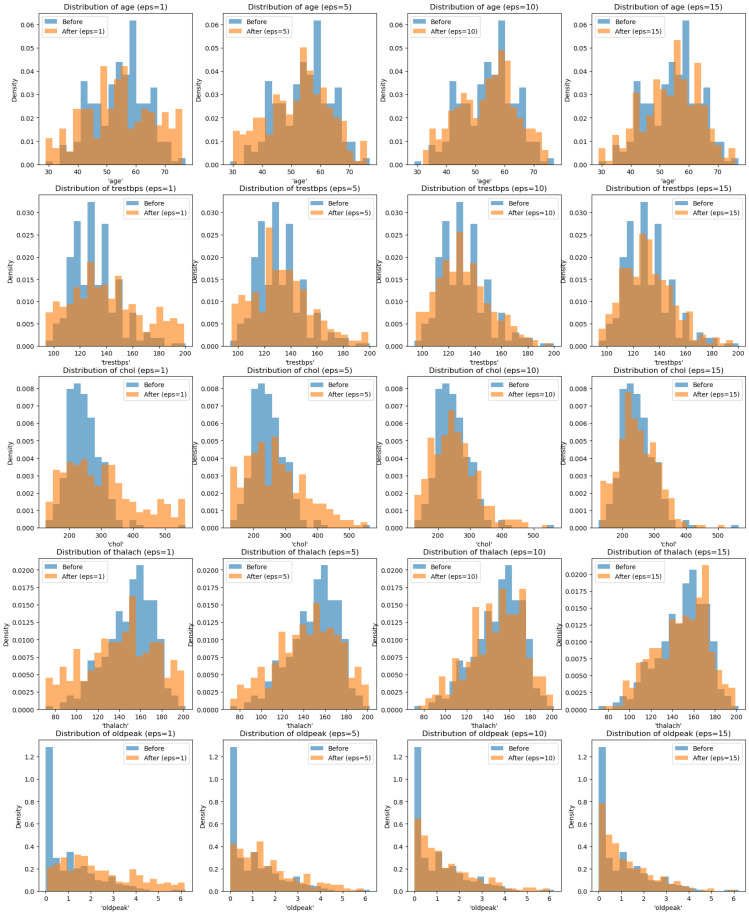
Privatize version analysis on the Angiographic Coronary Disease dataset.

For simplicity, the bounds in our experiments followed the minimum and maximum values in the dataset. We also assumed that these bounds were based on public knowledge, and the contribution value was set to 1. Based on the results, there was a trend that, as the epsilon value increased, the distribution followed closely to the actual distribution. As the epsilon value decreased, the distribution flattened and the density of the tails slightly increased (based on Figure 11 when the epsilon value was 1).

The crucial factor is whether this technique effectively privatizes the dataset. As we know, a measurement is ε-differentially private when, for every pair of adjacent datasets *u* and *v*, $\mathrm{Pr}\left[M(u)\in S\right]\leq e^{\varepsilon }\;\cdot \;\mathrm{Pr}\left[M(v)\in S\right]$ applies. To the best of our knowledge, there is no formal definition of a private dataset. However, we propose extending this definition to the entire dataset such that, for every pair of adjacent datasets *u* and *v* that differ by a single row, the dataset is ε-differentially private when $\mathrm{Pr}\left[d_{u}\in D\right]\leq e^{\varepsilon }\;\cdot \;\mathrm{Pr}\left[d_{v}\in D\right]$, where *D* refers to the universal dataset that contains all the possible rows of data, $d_{u}$ is the dataset that we are trying to privatize, and $d_{v}$ is its adjacent dataset. This definition has yet to be proven correct, but it is our best attempt to formalize the concept of an ε-differentially private dataset.

Based on Figure 11, we observed that the greater the epsilon value, the closer the distribution of the privatized dataset was to the actual dataset containing sensitive data. This suggests that the method approached the concept of a private dataset that was as defined earlier. As the epsilon value increased to *∞*, the distribution was expected to closely resemble the actual dataset as there would be no privacy guarantee. Thus, this method may be useful in specific situations for privatizing an entire dataset.

However, this version has its limitations. The first limitation is that the privatize version only works for numerical data. Many datasets, such as the Adult Census Income dataset, contain categorical data. This limits the functionality of this version, as it does not provide any privacy guarantees for datasets with a large amount of categorical data. In the worst-case scenario, the privatize version cannot privatize datasets that are composed entirely of categorical data.

Moreover, the method of privatizing the dataset does not provide much utility. Each numerical data point is independently privatized of the others, which means the resulting dataset may lack meaningful relationships between data points. If this dataset is used to train machine learning models or artificial intelligence, the privatized version may teach incorrect relationships. For instance, consider Figure 11, if the model is trained on the true dataset, it learns the correct relationships between the data points. However, after privatizing with an epsilon value of 1, the model learns relationships based on the privatized dataset, which is represented by the orange color. This altered relationship may lead to incorrect predictive behavior due to the discrepancies between the true relationships and those learned from the privatized data. Although the noise is calibrated, it does not account for the relationships between data points within the dataset. However, if the data points are independent of each other, this version may still be effective.

### 5.5. Application Analysis and Its Limitations

The web application was designed with an instructional section on the left that explains how differential privacy works, while the right section allows users to interact with the application. However, the application has some limitations. Although instructions are provided, they are primarily in textual form without visual cues to indicate the exact locations of functionalities. Additionally, there are no tooltips or hover text to further assist users in understanding the application’s features.

The “query” version of the application is useful as it provides users with the flexibility to test different queries. However, it currently supports only five types of queries: count, max, min, sum, and average. It lacks functionality for other important queries, such as quantiles and medians, indicating limited capabilities in its initial stages. Moreover, the queries, such as the minimum query, lack flexibility as they do not allow users to specify a range for the querying.

Regarding the privatize version, the application displays the original and privatized datasets, enabling users to compare them. However, this version has not been fully validated and can only be used in specific circumstances where each attribute in the dataset is independent. Furthermore, despite the various mechanisms and variants available in the literature, the application currently only utilizes the Laplace mechanism.

Moreover, the application currently lacks sample datasets, such as the Adult Census Income dataset and the Angiographic Coronary Disease dataset, for users to experiment with. Users must manually download “csv” files from the internet and upload them to the website, which is inconvenient. Additionally, the application only supports the “csv” file format, demonstrating a lack of flexibility. Furthermore, there is no functionality to display data distributions, which would help users better understand the dataset.

Additionally, the application has several more limitations on the technical side. Firstly, the epsilon values for maximum and minimum queries are not proportionate to what is typically found in the literature. In order to have similar output distributions as an epsilon value of 1, the maximum and minimum queries have to use large epsilon values like 500. Our experiments also indicate that large numbers are still not accepted, potentially providing inaccurate information to users.

## 6. Conclusions and Future Work

This paper introduced a web application that is aimed at making the complex principles of differential privacy accessible to a wider audience. The application offers two key features: a query version demonstrating differential privacy for statistical queries, and a privatize version applying DP techniques to entire datasets. Through these implementations, we identified discrepancies between the OpenDP library’s handling of maximum and minimum queries and theoretical expectations, highlighting critical areas for refinement in current DP tools. Moreover, we introduced a foundational framework for dataset privatization, which, while in its early stages, lays the groundwork for more robust privacy-preserving methods. Despite its limitations, including the restricted range of queries and current focus on numerical attributes, this application provides a valuable tool for bridging the gap between DP theory and practice. Future work will address these limitations by expanding query options, incorporating categorical data, and enhancing usability through an improved interface. By exposing discrepancies in library implementations and providing practical solutions, this work contributes to advancing the implementation and accessibility of differential privacy mechanisms.

## Figures and Tables

**Table 1:** Summary of the different epsilon values in real-world applications.

Company	Use Cases	Epsilon/Epsilon and Delta Values
Apple	QuickType Suggestion	ϵ=16
	Emoji Suggestion	ϵ=4
	Lookup Hints	ϵ=8
	Health Type Usage	ϵ=2
	Safari Energy Draining Domains and Safari Crashing Domains	ϵ=8
	Safari Autoplay Intent Detection	ϵ=16
Facebook	Full URL Dataset	99% of users: ϵ=0.41 and $\delta =10^{-5}$, 96.6% of users: ϵ=1.69 and $\delta =10^{-5}$
	Movement Range Maps	ϵ=2
Google	Community Mobility Reports	ϵ=2.64
	Environmental Insights Explorer	ϵ=2
	Gboard next-word prediction models	$\delta =10^{-5}$ and ϵ varying between 0.69 and 10.61 depending on the language
	Gboard out-of-vocabulary word discovery	ϵ=0.32 and $\delta =10^{-10}$
	Search Trends Symptoms Dataset	ϵ=1.68
	Google Shopping	ϵ=1 and $\delta =10^{-9}$
	Google Trends	ϵ=2 and $\delta =10^{-10}$
	Urban Mobility Data	ϵ=0.66 and $\delta =2.1\times 10^{-29}$
	Vaccination Search Insights	ϵ=2.19 and $\delta =10^{-5}$
Israel’s Ministry of Health	Synthetic Dataset of live births in 2014 in Israel	ϵ=9.98
LinkedIn	Audience Engagements API	ϵ=34.9 and $\delta =7\times 10^{-9}$
	Labor Market Insights (Lists the companies who are hiring most)	ϵ=14.4 and $\delta =1.2\times 10^{-9}$
	Labor Market Insights (Job titles that most people are being hired for)	ϵ=14.4 and $\delta =1.2\times 10^{-9}$
	Labor Market Insights (Most popular skills for the jobs)	ϵ=0.3 and $\delta =3\times 10^{-10}$
	Race and Ethnicity of Users	ϵ=4.5
Microsoft	Global Victim-Perpetrator Synthetic Dataset	ϵ=12 and $\delta =5.8\times 10^{-6}$
	Telemetry collection in Windows	ϵ=1.672
	U.S. Broadband Coverage Dataset	ϵ=0.2
OhmConnect	Energy Differential Privacy project	ϵ=4.72 and $\delta =5.06\times 10^{-9}$
United States Census Bureau	County Business Patterns	ϵ=34.92 and $\delta =10^{-5}$
	2020 Census (Redistricting Data)	ϵ=13.64 and $\delta =10^{-5}$
	2020 Census (Demographic Housing and Characteristics File (DHC))	ϵ=19.46 and $\delta =10^{-5}$ (for counting people) ϵ=25.87 and $\delta =10^{-5}$ (for counting households)
	2020 Census (Detailed DHC-A)	ϵ=49.21 and $\delta =10^{-5}$
	2020 Census (Detailed DHC-B)	ϵ=45.68 and $\delta =10^{-5}$
	Post-Secondary Employment Outcomes	ϵ=3
Wikimedia Foundation	Statistics about how many distinct users visited each Wikipedia page on each day, from each country (from 1 July 2015 to 8 February 2017)	ϵ=1
	Statistics about how many distinct users visited each Wikipedia page on each day, from each country (9 February 2017 to 5 February 2023)	ϵ=1
	Statistics about how many distinct users visited each Wikipedia page on each day, from each country (6 February 2023 onwards)	ϵ=0.72 and $\delta =10^{-5}$
	Statistics about editor activity by project and country, on Wikipedia and other Wikimedia projects (published monthly)	ϵ=2
	Statistics about editor activity by project and country, on Wikipedia and other Wikimedia projects (published weekly)	ϵ=2
	Statistics about editor activity by project and country, on Wikipedia and other Wikimedia projects (A one-off release for Russian editors)	ϵ=0.1

## Data Availability

Data are contained within the article.

## References

[1] Christl W., Spiekermann S. (2016). Networks of Control a Report on Corporate Surveillance, Digital Tracking, Big Data & Privacy.

[2] Invisibly (2021). 7 Examples of Data Misuse in the Modern World. https://www.invisibly.com/realtime-research/.

[3] Cadwalladr C., Graham-Harrison E. (2018). Revealed: 50 million facebook profiles harvested for cambridge analytica in major data breach. The Guardian.

[4] O’Neill J. (2023). PSNI: Major Data Breach Identifies Thousands of Officers and Civilian Staff. https://www.bbc.com/news/uk-northern-ireland-66445452.

[5] Sharkey K. (2024). PSNI Could Be Fined £750,000 over Data Breach. https://www.bbc.com/news/articles/czqqjglq1lyo.

[6] Perez E., Cohen Z., Herb J., Bertrand N., Liptak K. (2023). FBI Arrests 21-Year-Old Air Force Guardsman in Pentagon Leak Case|CNN Politics. https://edition.cnn.com/videos/world/2023/04/14/exp-fbi-arrest-jack-teixeira-fst-041412aseg1-cnni-world.cnn.

[7] Maruta J., Cho C., Raphan T., Yakushin S.B. (2024). Symptom reduction in mal de débarquement syndrome with attenuation of the velocity storage contribution in the central vestibular pathways. Front. Rehabil. Sci..

[8] intersoft Consulting General Data Protection Regulation GDPR. https://gdpr-info.eu/.

[9] Federal Court of Australia Guide to the Anonymisation of Personal and Sensitive Information. https://www.fedcourt.gov.au/online-services/preparing-documents-for-the-court/guide-to-the-anonymisation.

[10] Hamsanandhini S., Balasubramanie P. (2024). IoT Data Encryption and Phrase Search-Based Efficient Processing Using a Fully Homomorphic-Based SE (FHSE) Scheme. Pervasive Mob. Comput..

[11] Vaiwsri S., Ranbaduge T., Christen P. (2024). Encryption-based sub-string matching for privacy-preserving record linkage. J. Inf. Secur. Appl..

[12] Mahato G.K., Banerjee A., Chakraborty S.K., Gao X.Z. (2024). Privacy preserving verifiable federated learning scheme using blockchain and homomorphic encryption. Appl. Soft Comput..

[13] Damien Desfontaines (2021). A List of Real-World Uses of Differential Privacy. Ted Is Writing Things (Personal Blog). https://desfontain.es/blog/real-world-differential-privacy.html.

[14] Sweeney L. (2002). k-Anonymity: A Model for Protecting Privacy. Int. J. Uncertain. Fuzziness-Knowl.-Based Syst..

[15] Machanavajjhala A., Kifer D., Gehrke J., Venkitasubramaniam M. (2007). *L*-diversity: Privacy beyond k-anonymity. ACM Trans. Knowl. Discov. Data.

[16] Li N., Li T., Venkatasubramanian S. t-Closeness: Privacy Beyond k-Anonymity and l-Diversity. Proceedings of the 2007 IEEE 23rd International Conference on Data Engineering.

[17] Dwork C., Bugliesi M., Preneel B., Sassone V., Wegener I. (2006). Differential Privacy. Automata, Languages and Programming.

[18] Dwork C., Roth A. (2014). The Algorithmic Foundations of Differential Privacy. Found. Trends Theor. Comput. Sci..

[19] Rajendran K., Jayabalan M., Rana M.E. (2017). A study on k-anonymity, l-diversity, and t-closeness techniques. IJCSNS.

[20] Meyerson A., Williams R. On the complexity of optimal K-anonymity. Proceedings of the Twenty-Third ACM SIGMOD-SIGACT-SIGART Symposium on Principles of Database Systems.

[21] Aggarwal C.C. On k-anonymity and the curse of dimensionality. Proceedings of the 31st VLDB Conference.

[22] Ji Z., Lipton Z.C., Elkan C. (2014). Differential Privacy and Machine Learning: A Survey and Review. arXiv.

[23] Abadi M., Chu A., Goodfellow I., McMahan H.B., Mironov I., Talwar K., Zhang L. Deep Learning with Differential Privacy. Proceedings of the 2016 ACM SIGSAC Conference on Computer and Communications Security.

[24] Ponomareva N., Hazimeh H., Kurakin A., Xu Z., Denison C., McMahan H.B., Vassilvitskii S., Chien S., Thakurta A.G. (2023). How to DP-fy ML: A Practical Guide to Machine Learning with Differential Privacy. J. Artif. Intell. Res..

[25] Near J.P., Abuah C. (2021). Programming Differential Privacy. https://programming-dp.com/book.pdf.

[26] Dwork C., Kenthapadi K., McSherry F., Mironov I., Naor M., Vaudenay S. (2006). Our Data, Ourselves: Privacy Via Distributed Noise Generation. Proceedings of the Advances in Cryptology—EUROCRYPT.

[27] Mironov I. (2017). Rényi differential privacy. Proceedings of the 2017 IEEE 30th computer security foundations symposium (CSF).

[28] Bun M., Steinke T. (2016). Concentrated Differential Privacy: Simplifications, Extensions, and Lower Bounds. arXiv.

[29] Dwork C., Rothblum G.N. (2016). Concentrated Differential Privacy. arXiv.

[30] Kasiviswanathan S.P., Lee H.K., Nissim K., Raskhodnikova S., Smith A. (2010). What Can We Learn Privately?. arXiv.

[31] Mironov I. On significance of the least significant bits for differential privacy. Proceedings of the 2012 ACM Conference on Computer and Communications Security.

[32] Differential Privacy Team (2020). Secure Noise Generation. https://github.com/google/differential-privacy/blob/main/common_docs/Secure_Noise_Generation.pdf.

[33] Canonne C.L., Kamath G., Steinke T., Larochelle H., Ranzato M., Hadsell R., Balcan M., Lin H. (2020). The Discrete Gaussian for Differential Privacy. Advances in Neural Information Processing Systems.

[34] Jin J., McMurtry E., Rubinstein B.I.P., Ohrimenko O. Are We There Yet? Timing and Floating-Point Attacks on Differential Privacy Systems. Proceedings of the 2022 IEEE Symposium on Security and Privacy (SP).

[35] Narayanan A., Shmatikov V. (2006). How To Break Anonymity of the Netflix Prize Dataset. arXiv.

[36] Holohan N. (2017). Mathematical Foundations of Differential Privacy. Ph.D. Thesis.

[37] Fartale H. (2023). A Survey of Differential Privacy Frameworks. https://blog.openmined.org/a-survey-of-differential-privacy-frameworks/.

[38] Detrano R.C., Jánosi A., Steinbrunn W., Pfisterer M.E., Schmid J.J., Sandhu S., Guppy K., Lee S., Froelicher V. (1989). International application of a new probability algorithm for the diagnosis of coronary artery disease. Am. J. Cardiol..

[39] Becker B., UCI Machine Learning Kaggle Team (2016). Adult Census Income. https://www.kaggle.com/datasets/uciml/adult-census-income?resource=download.

[40] Morgan J. Identifying Categorical Data. https://jeffreymorgan.io/articles/identifying-categorical-data/.

[41] OpenDP A Framework to Understand DP. https://docs.opendp.org/en/stable/index.html.

[42] OpenDP Working with Unknown Dataset Sizes. https://docs.opendp.org/en/stable/examples/unknown-dataset-size.html.

[43] Penner E. Total Cholesterol: 2.4 mmol/L (93 mg/dL). https://www.elo.health/biomarkers/total-cholesterol-overview/24/.

[44] Penner E. Total Cholesterol: 982 mg/dL. https://www.elo.health/biomarkers/total-cholesterol-overview/982/.

